# Refractory Status Epilepticus Following Erroneous Intrathecal Injection of Tranexamic Acid: A Case Report and Long‐Term Follow‐Up and Narrative Review

**DOI:** 10.1155/cria/1049073

**Published:** 2026-10-08

**Authors:** Hamza Yousfi, Leda Nobile, Nicolas Gaspard, Fabio Taccone

**Affiliations:** ^1^ Department of Intensive Care Hôpital Universitaire de Bruxelles (HUB) Erasme Hospital Université Libre de Bruxelles (ULB) Brussels Belgium ulb.ac.be; ^2^ Department of Neurology Hôpital Universitaire de Bruxelles (HUB) Erasme Hospital Université Libre de Bruxelles (ULB) Brussels Belgium ulb.ac.be; ^3^ Department of Neurology Yale University School of Medicine New Haven Connecticut USA yale.edu

**Keywords:** case report, intrathecal injection, posttraumatic stress disorder, rhabdomyolysis, spinal anesthesia, status epilepticus, tranexamic acid

## Abstract

**Introduction:**

Accidental intrathecal injection of tranexamic acid (TXA) is a rare but catastrophic preventable adverse event associated with high mortality and severe neurological consequences. It represents a major neurological emergency that requires immediate specialized management.

**Case Presentation:**

A 32‐year‐old man erroneously received an intrathecal injection of 300 mg of TXA instead of bupivacaine during a saphenous vein stripping procedure. He rapidly developed refractory myoclonic status epilepticus, sympathetic hyperactivity, and rhabdomyolysis. Management included multiple antiseizure medications, continuous EEG‐guided sedation, and targeted use of neuromuscular blockade. The patient recovered without immediate neurological deficit but developed persistent cognitive impairment and posttraumatic stress disorder at follow‐up.

**Conclusions:**

This case highlights the severe neurotoxicity of intrathecal TXA and the importance of early and aggressive intensive care management. It also underscores the need for long‐term multidisciplinary follow‐up for the assessment of anesthetic procedure‐related complications.

## 1. Introduction

Tranexamic acid (TXA), a synthetic antifibrinolytic developed in the 1960s [1], has become a cornerstone in the management of perioperative and traumatic bleeding [2–4] and is widely used to reduce blood loss and the need for transfusion, particularly in orthopedic [2], cardiac [5], and obstetric [6, 7] surgery, as well as to reduce the risk of mortality in trauma patients (CRASH‐2 and CRASH‐3 trials) [3, 4]. Although TXA has a generally favorable safety profile, accidental intrathecal administration may lead to severe and occasionally fatal neurological complications [8, 9]. Such events are typically the result of medication errors, the incidence of which in anesthesia ranges from 1 in 130 to 1 in 900 procedures [10–12]. While uncommon, these errors are responsible for serious adverse outcomes in nearly 7% of cases [13].

Regional anesthesia, whether neuraxial or peripheral, poses a particular risk [13, 14]. Approximately one‐quarter of reported anesthesia‐related errors involve intrathecal injection of drugs intended for another route, often due to confusion caused by similar‐looking vials [13, 14]. When TXA is involved, prompt and aggressive management is required, including deep sedation, precise titration of antiseizure medications (ASMs), and continuous electroencephalography (cEEG) monitoring [9, 15, 16].

We reported the case of a 32‐year‐old patient who was the victim of such an error. This case highlights not only the importance of rapid initial intervention but also the need for prolonged neurological follow‐up to assess potential long‐term sequelae.

## 2. Case Presentation

A 32‐year‐old male patient with no significant past medical or surgical history and classified as the American Society of Anesthesiologists II [17] was scheduled for a right great saphenous vein stripping procedure combined with phlebectomies as treatment for symptomatic and decompensated chronic venous insufficiency. His medical history included active tobacco use, occasional cannabis consumption, and no known drug allergies. A preoperative SARS‐CoV‐2 PCR test performed on a nasopharyngeal swab was negative. The remaining blood workup, including complete blood count, inflammatory markers, coagulation profile, fluid and electrolyte balance, and renal and hepatic function, was strictly within normal limits.

Premedication consisted of 15 mg of prazepam. Spinal anesthesia was planned at the L4‐L5 level using 3 mL of 0.5% hyperbaric bupivacaine; in parallel, 1 g of paracetamol and 10 mg of dexamethasone were administered intravenously. Approximately ten minutes after the local anesthetic was administered, the patient developed sudden and severe lower back pain, rapidly followed by intense cramping in the lower limbs. He presented with tachycardia (170 bpm) and hypertension (165/80 mmHg), while initial clonic movements of the lower limbs rapidly progressed to generalized tonic‐clonic seizures, accompanied by a loss of consciousness. Midazolam 5 mg was administered immediately with no notable effect. Given the rapid clinical deterioration, the decision was made to secure the airway by orotracheal intubation, followed by the initiation of mechanical ventilation. An additional 5 mg of midazolam and 100 mg of propofol were administered, followed by continuous sedation with propofol. Persistent seizures led to the administration of a 2‐g loading dose of levetiracetam. At that point, the discovery of an empty ampoule of TXA on the anesthesia tray, next to an intact ampoule of bupivacaine, confirmed the accidental intrathecal administration of 300 mg of TXA (3 mL of a 10% solution).

Due to ongoing myoclonic activity, a loading dose of 2400 mg of valproic acid was administered in combination with continuous sedation using propofol (4 mg/kg∗h) and midazolam (20 mg∗h). The initial neurological examination, performed under sedation, revealed bilaterally reactive, symmetric miotic pupils, bilateral plantar reflexes in flexion, and brief myoclonic jerks of all four limbs in response to stimulation. Initial arterial blood gas analysis showed mixed acidosis (pH 7.05), with a respiratory (PaCO_2_ 49 mmHg) and a metabolic component (lactate levels at 13.6 mmol/L), the latter probably due to ictal motor activity, with no associated electrolyte abnormalities. A contrast‐enhanced brain CT scan showed no evidence of acute intracranial lesions. No brain MRI was performed during the acute episode.

Due to the need for cEEG monitoring and individualized titration of ASMs, the patient was transferred to our institution. The clinical picture showed ongoing seizures despite two appropriately administered treatment lines, consistent with refractory status epilepticus (RSE) [16], requiring intensive management under continuous sedation with propofol and midazolam [16]. A blood panel performed 6 h after seizure onset revealed Stage I acute kidney injury, according to Kidney Disease Improving Global Outcomes criteria [18], with a transient rise in serum creatinine to 1.31 mg/dL (from a baseline of 0.95 mg/dL) and blood urea at 32.4 mg/dL. This renal impairment resolved rapidly with hydration in less than 24 h. There was neutrophil‐predominant hyperleukocytosis (17,240/mm^3^, including 15,950 neutrophils/mm^3^) without significant elevation of C‐reactive protein (0.5 mg/L). Creatine kinase (CK) levels were elevated at 1036 IU/L. The remainder of the laboratory workup, including coagulation profile, electrolyte balance, and liver function, was unremarkable. In parallel, ceftriaxone (2 g twice daily for 5 days) was administered as antibiotic prophylaxis because a potential breach in aseptic conditions could not be excluded in the context of the medication error. No lumbar puncture or CSF analysis was performed specifically to exclude meningitis before antibiotic discontinuation, and no clinical features suggestive of meningitis developed during hospitalization.

The initial cEEG recording showed a burst‐suppression pattern associated with massive reflex myoclonus (RM) and stimulus‐induced highly epileptiform bursts, predominantly in anterior regions. Due to persistent epileptic manifestations, midazolam was gradually increased to 40 mg/h, and neuromuscular blockade with cisatracurium was initiated. Under this intensive regimen, both clinical and electroencephalographic ictal activity progressively improved. After 12 h, the burst‐suppression pattern resolved. The cEEG then showed a continuous background with signs of sedation but without any associated epileptiform discharges.

Propofol and cisatracurium were discontinued, with no recurrence of epileptiform activity or myoclonus on midazolam infusion at 40 mg/h, combined with levetiracetam at 1500 mg twice daily. Given this favorable progression, midazolam was gradually tapered over 72 h under cEEG guidance. The patient was successfully weaned from mechanical ventilation and extubated 4 hours after midazolam discontinuation without any complications.

Upon awakening, the patient was alert and oriented. Cognitive examination revealed no coarse deficit in speech, memory, or attention. Motor and sensory examination was normal, with symmetrical deep tendon reflexes and bilateral plantar flexor responses. The patient had no recollection of events during intubation and sedation. He was informed of the adverse event and subsequent clinical evolution.

On Day 5, the patient developed severe rhabdomyolysis, confirmed by markedly elevated muscle biomarkers, CK reached 63,645 IU/L, AST 1194 IU/L, and LDH 1822 IU/L. This complication was resolved with hyperhydration. On Day 7, the patient was transferred from the ICU to the neurology ward. His condition remained stable, allowing discharge from the hospital 11 days after admission. At discharge, levetiracetam (1 g twice daily) was progressively tapered by 500 mg per week, with complete discontinuation after 4 weeks.

Over the long term, neurological and neuropsychological follow‐up documented persistent concentration and memory difficulties. Formal neuropsychological assessment demonstrated impaired resistance to interference in working memory (Brown–Peterson test: 72.2% at 5 s, *t* = −2.14; 61.1% at 10 s, *t* = −2.26; 33.3% at 20 s, *t* = −3.89), impaired verbal episodic memory (RL15: mean words recalled 10.4, *t* = −2.36; consistency index 44.23, *t* = −2.81; 30 min delayed recall 12, *t* = −3.41), and impaired oral naming (Lexis 80: 69/80, *t* = −2.81 without phonemic cueing and *t* = −2.92 with phonemic cueing). Inhibition, cognitive flexibility, and overall planning abilities were preserved. The patient also experienced chronic headaches, as well as muscle and lower back pain, which were managed with physiotherapy. A brain MRI performed 6 months after the episode of RSE showed bilateral claustral hyperintensity on T2/FLAIR sequences, which had completely resolved on follow‐up MRI 6 months later. Finally, a posttraumatic stress disorder (PTSD) was diagnosed, attributed to the medication error and the ICU hospitalization, requiring specialized and prolonged psychological support.

## 3. Discussion

This case illustrates the severity of accidental intrathecal administration of TXA, a rare but devastating medication error reported in many countries and associated with severe neurological and cardiovascular complications [8, 9, 19]. It is responsible for status epilepticus (SE) in over 85% of reported cases [9]. Although rare, this error is associated with a reported mortality rate between 36% and 50% and may be accompanied by severe sympathetic hyperactivity, potentially leading to ventricular arrhythmias or cardiac arrest within hours of injection [8, 9, 19]. In our case, RSE was further complicated by severe rhabdomyolysis. In this case, we observed a two‐phase evolution: an initial full neurological recovery followed by persistent neuropsychological deficits; whether this could be directly linked to the intrathecal TXA administration or to PTSD is yet to be elucidated. Delayed complications following erroneous intrathecal TXA administration remain underreported in the literature [8, 9].

A focused literature search was conducted in PubMed/MEDLINE using the following search strategy: (“spinal anaesthesia” OR “spinal anesthesia” OR “intrathecal” OR “epidural”) AND (“tranexamic acid” OR “medication errors”). The search was supplemented by Google Scholar using the terms “intrathecal tranexamic acid,” “epidural tranexamic acid,” and “neuraxial tranexamic acid,” as well as by manual screening of the reference lists of relevant publications. Publications available up to February 1, 2026, were considered. For the comparison of clinical cases, reports describing inadvertent intrathecal TXA administration with a clearly documented route of administration and clinical course or outcome were included. The identified literature was narratively reviewed to compare previously reported cases with the present case and to contextualize the pathophysiology, clinical management, neurological outcomes, and prevention of inadvertent intrathecal TXA administration.

Despite its generally favorable safety profile, TXA poses a significant neurotoxic risk even when administered intravenously, particularly among patients undergoing cardiac surgery [15]. TXA induces acute neurotoxicity by interfering with inhibitory neurotransmission within the central nervous system [20]. It acts as a competitive antagonist of GABAergic and glycinergic receptors at spinal and cortical levels, shifting the excitation‐inhibition balance toward neuronal hyperexcitability [20–23]. The clinical sequence observed in our patient, i.e., sudden back pain, cramps, myoclonus, and generalized seizures, closely mirrors those reported in the literature [9, 24]. Sudden back pain may be explained by TXA‐induced spinal disinhibition of sensory neurons in the dorsal horn through blockade of glycine and GABA_A_ receptors, leading to exaggerated activation of nociceptive pathways [20, 22, 23, 25]. At the cortical level, TXA blocks GABA_A_ receptors in epileptogenic zones, facilitating synchronous neuronal discharges and seizure onset [20–23]. Experimental models confirm that intrathecal or subdural TXA produces dose‐dependent epileptiform activity [15, 26], consistent with our patient’s rapid evolution to generalized and refractory SE [27].

The 300‐mg intrathecal dose administered in our patient represents an intermediate‐to‐high dose within the range reported in previous cases, which has varied from 50 to 500 mg [8, 9]. However, the relationship between dose and clinical outcome appears inconsistent. Severe neurotoxicity, including RSE and multiorgan dysfunction, has been reported after doses as low as 50 mg, while fatal outcomes have occurred after doses of 160–200 mg, and nonfatal outcomes have also been documented after doses of 300–350 mg [8, 9]. Thus, although the dose received by our patient was substantial, current case‐based evidence does not support a clear dose–outcome relationship.

Because no antidote exists and no standardized therapeutic protocol has been validated for intrathecal TXA toxicity, management remains largely supportive and symptomatic, with priorities including immediate airway protection, aggressive seizure control, hemodynamic stabilization, continuous EEG‐guided titration of ASMs, and anticipation of systemic complications such as ventricular arrhythmias, metabolic derangements, and rhabdomyolysis [9]. In our case, seizure control was achieved within 12 h using an aggressive pharmacologic strategy, including propofol, midazolam, valproate, and levetiracetam, while cEEG monitoring was continued for 4 days to guide real‐time titration of ASMs. This approach is consistent with the current international recommendations, which emphasize the importance of early continuous sedation in RSE with prominent motor manifestations, as well as the systematic use of cEEG to detect nonconvulsive seizures and guide dynamic treatment adjustment [16, 28]. Compared with published cases, our management was notable for both the rigor of seizure management and the extended duration of cEEG monitoring. Goyal et al. reported an RSE case initially resistant to benzodiazepines, ultimately requiring barbiturates, with intermittent EEG monitoring [29]. Prior et al. achieved seizure suppression with pentobarbital, without prolonged EEG monitoring [30]. Godec et al. initiated a combination of ventriculolumbar lavage and sevoflurane anesthesia, but EEG monitoring remained brief [31]. In addition, cerebrospinal fluid (CSF) lavage has been reported in several published cases, and recent narrative reviews suggest that early CSF lavage may offer outcome benefits by reducing intrathecal TXA exposure, although the evidence remains limited to case reports [8, 9, 32–34]. Thus, although CSF lavage was not performed in our patient, it might reasonably have been considered as an adjunctive measure. In line with the current recommendations, our case supports the potential benefit of early integration of prolonged cEEG monitoring in intrathecal TXA‐associated RSE, particularly to monitor seizure activity and guide the titration of ASMs and sedative therapy [9, 16, 28].

Our patient showed a refractory RM, despite the use of midazolam and propofol, which prompted the early initiation of continuous cisatracurium infusion. The persistent RM likely reflects disinhibition of motor circuits in the brainstem and spinal cord [22, 25, 26]. By antagonizing glycine receptors, TXA impairs inhibitory control over reflex pathways, resulting in massive RM resistant to sedation. Although rarely documented in the literature [32–35], continuous infusion of a neuromuscular blocking agent resulted in rapid control of myoclonus and allowed for better titration of ASMs while reducing muscle injury associated with rhabdomyolysis. Thus, the use of neuromuscular blocking agents, combined with sedation, seems to be an effective strategy to manage refractory RM not controlled by sedation alone and to prevent complications of prolonged SE, as also supported by recent discussions on strategies to reduce morbidity in the ICU [9].

Our patient developed severe rhabdomyolysis; this complication is rarely reported in similar cases. To our knowledge, only Akodjenou et al. described a comparable case of rhabdomyolysis within 48 h associated with multiple organ failure and transient paraplegia [36]. In our case, rhabdomyolysis was likely attributable to the intensity and duration of the initial tonic‐clonic seizures, as well as persistent RM, both causing prolonged muscle contractions. Early management included intravenous hyperhydration and neuromuscular blockade, with the aim to minimize the severity of tubular precipitation of muscular enzymes, in particular myoglobin [37]. Ceftriaxone was administered prophylactically because the circumstances surrounding the medication error raised concern for a possible breach in the aseptic technique. This was a case‐specific decision rather than an evidence‐based prophylactic strategy. Given the lack of data supporting routine antibiotic prophylaxis after inadvertent intrathecal TXA administration, we do not advocate its systematic use. However, the potential risk of iatrogenic CNS infection should be assessed on an individual basis when sterility may have been compromised, with further diagnostic evaluation and antimicrobial therapy guided by the clinical context.

Unlike most published cases, which tend to evolve either toward rapid fatality [38, 39] or favorable short‐term outcomes [40–42], our patient required more than 2 years of outpatient follow‐up because of persistent cognitive and psychological sequelae. The patient reported having chronic, low‐grade but persistent headaches with no identified cause, memory and attention problems, severe fatigue, and serious sleep issues. He also described panic attacks triggered by specific environments, such as hospitals, as well as heightened sensory sensitivity to sounds and sights, and episodes of intense anxiety or dissociation. These episodes were marked by mental and physical shutdowns, tingling in the limbs and overwhelming stress. These symptoms greatly impacted his quality of life and ability to function. Consequently, the patient had to stop his musical practice due to problems with memory and concentration, and he abandoned a culinary training position because of fatigue, cognitive challenges, and difficulty tolerating overstimulating settings. He also reported difficulties engaging in social activities, especially in noisy places, which contributed to considerable disability in his daily and professional life. All of these symptoms are consistent with PTSD [43], but it should be investigated whether organic damage caused by intrathecal administration of TXA could be responsible for long‐term neuropsychological and cognitive impairment [44]. The bilateral claustral T2/FLAIR hyperintensity observed on MRI 6 months after the episode of RSE may resemble the claustrum sign previously described in association with RSE. However, its first documentation at 6 months is atypical, as Meletti et al. reported claustral abnormalities occurring a mean of 10 days after SE onset (range: 3–25 days), with resolution in most cases during follow‐up, although persistence up to 12 months was observed in one patient [45]. Broadly, peri‐ictal MRI abnormalities associated with SE may be reversible or persist beyond the acute phase; in a recent prospective cohort, peri‐ictal MRI abnormalities persisted in 16% of patients at 4‐week follow‐up, while long‐term structural changes were observed in a substantial proportion of patients [46]. In our patient, no brain MRI was performed during the acute episode, which represents a major limitation and precludes determining whether the 6‐month claustral hyperintensity reflected persistence of an earlier SE‐related lesion, a delayed manifestation, or a nonspecific structural abnormality. In the absence of another documented structural explanation in this case, an SE‐related process remains a plausible interpretation of the finding. However, the complete resolution of the abnormality on follow‐up MRI at 12 months, while compatible with a reversible process, does not by itself establish a causal relationship with the previous episode of RSE. Regardless of the underlying mechanism, the substantial and lasting burden of such an event underscores the importance of continued multidisciplinary care. To our knowledge, this is the first report to provide such a detailed and long‐term neurocognitive and psychological follow‐up of a survivor of TXA‐induced RSE.

This case underscores that intrathecal TXA toxicity is not only a neurological emergency but also a major medication‐safety failure. Reported cases continue to emerge from multiple countries and practice environments, suggesting persistent international vulnerability and probable underreporting [9, 47]. Broadly, Viscusi et al., in a review of 133 cases of wrong‐route medication administration via neuraxial or peripheral routes, found that 25.4% involved erroneous intrathecal administration of a drug intended for another route, with several confirmed cases involving TXA [14].

Errors related to intrathecal TXA administration are multifactorial. The most commonly identified cause is vial confusion—particularly between TXA and hyperbaric bupivacaine—due to substantial visual similarity, which accounts for over 90% of reported errors [9, 48]. Other contributing factors include unsafe storage, poorly distinguishable labeling, nonstandardized drug preparation during spinal anesthesia, inadequate communication regarding changes in drug supply, and the absence of independent double‐checking [9, 47, 48]. Additional systemic and human factors include insufficient training or supervision, lack of protocols and error reporting systems, and failures of situational awareness [9, 47, 48].

Prevention, therefore, requires a multidisciplinary approach involving anesthesiologists, nurse anesthetists or assistants, pharmacists, operating‐room leadership, hospital safety teams, and manufacturers [47]. Practical measures include strict physical separation of TXA and local anesthetic storage, clear syringe labeling, communication of any change in product brand, packaging, or local storage practices to frontline staff, and implementation of independent double‐checks before any neuraxial injection [47, 48]. At the manufacturer level, redesign of look‐alike vials and more distinctive packaging may further reduce the risk [47]. At the individual level, vigilance alone is insufficient unless embedded in a robust process. The vial label should be checked when the drug is selected and again immediately before intrathecal administration; the operator should preferably prepare the neuraxial drug personally, and an independent double‐check should be structured and carried out without distraction [47]. The universal adoption of specific neuroaxial route connectors, such as NRFit^®^ connectors (ISO 80369‐6) is recommended [49]; these connectors are specifically designed to prevent wrong‐route administration by creating mechanical incompatibility between neuraxial and intravenous systems [48, 49]. Although this technical barrier is simple and effective, it may not prevent vial misidentification and could still allow the intrathecal administration of incompatible drugs, even when prepared safely in a dedicated neuroaxial device. Consequently, thorough checklists, protocols, and staff training must be implemented to prevent any inadvertent intrathecal injection.

## 4. Conclusions

Accidental intrathecal administration of TXA is a critical neurological emergency often associated with a poor prognosis. Intensive management, cEEG monitoring, a multimodal ASM strategy, and early neuromuscular blockade may allow functional recovery. Prolonged multidisciplinary follow‐up should be considered to assess and address long‐term sequelae. The continued occurrence of inadvertent intrathecal TXA injections underscores the need for robust medication‐safety systems in anesthesia.

**Nomenclature**
ASMs: Antiseizure medications
AST: Aspartate aminotransferase
cEEG: Continuous electroencephalography
CSF: Cerebrospinal fluid
CK: Creatine kinase
EEG: Electroencephalography
ICU: Intensive care unit
LDH: Lactate dehydrogenase
MRI: Magnetic resonance imaging
NRFit^®^
: Neuraxial and regional connector (ISO 80369‐6)
PaCO_2_
: Arterial partial pressure of carbon dioxide
PCR: Polymerase chain reaction
PTSD: Posttraumatic stress disorder
RM: Reflex myoclonus
RSE: Refractory status epilepticus
SE: Status epilepticus
TXA: Tranexamic acid

## Author Contributions

All authors contributed to patient care and to manuscript preparation.

## Funding

No funding was received for this work.

## Disclosure

All authors read and approved the final manuscript.

## Consent

Written informed consent was obtained from the patient for publication of this case report and any accompanying information. The signed consent form is available for review by the Editor‐in‐Chief upon request.

## Conflicts of Interest

The authors declare no conflicts of interest.

## Patient Perspective

Thank you for sharing the manuscript with me. Even though much of the medical vocabulary is difficult for me to understand, reading it helped me make sense of what happened and organize an experience that still sometimes feels unreal. My last clear memory before the incident is traveling to the hospital early in the morning. After that, I only have very brief flashes: being in an operating room, the injection in my lower back, and then a sudden and abnormally intense pain at the injection site and down my legs. I immediately alerted the team and remember feeling panic. After that, there is nothing—no memory of the seizures, being intubated, the transfer by ambulance, or the days in the ICU. I am only aware of this period through what I have been told and what I later read in medical reports. What remains are vague impressions, like very strange dreams.

I woke up several days later in the ICU, connected to multiple machines in a dark and noisy environment. I was confused and unable to think clearly, and I struggled to speak and move. What affected me most was the uncertainty; I kept asking whether I would be able to walk again, speak normally, and recover as I was before, and no one could answer with certainty. I was also constantly afraid of having another seizure and not surviving it. After my transfer to the neurology ward and then discharge home, recovery was much longer and harder than I expected. I required physiotherapy, and I experienced severe fatigue, hypersensitivity to stimulation (lights and sounds), and episodes of anxiety. Administrative and financial difficulties made this period even more stressful, and I started regular psychotherapy, which I still continue.

Over time, some things improved, but I still have symptoms that significantly affect my daily life: near‐daily headaches, memory and concentration difficulties, fatigue, insomnia, and panic attacks. These difficulties have affected my social and professional life and forced me to give up certain activities, especially music, and to rethink my career plans. I feel deep frustration about an avoidable error and its consequences, and I remain anxious about undergoing another procedure in the future. At the same time, I am profoundly grateful to the teams who took care of me and provided follow‐up, for their professionalism, kindness, and support. I hope that sharing my experience can help prevent this from happening to others. (Statement provided by the patient and translated from French by the authors.)

## Supporting Information

Additional supporting information can be found online in the Supporting Information section.

## Supporting information


**Supporting Information** CARE checklist_Refractory Status Epilepticus Following Erroneous Intrathecal Injection of Tranexamic Acid a Case Report and Long‐Term Follow‐Up.

## Data Availability

Data are not publicly available due to patient privacy. Data may be available from the corresponding author on reasonable request.
