# Supplementary material for: Refractory Status Epilepticus Following Erroneous Intrathecal Injection of Tranexamic Acid: A Case Report and Long‐Term Follow‐Up and Narrative Review
Source: Case Rep Anesthesiol. 2026 Oct 8;2026:1049073. doi: 10.1155/cria/1049073 (PMC13646888; doi:10.1155/cria/1049073)
Supplement: Supplementary file 1 [file CRIA-2026-1049073-s001.docx]

|  |  | **CARE Checklist of information to include when writing a case report** |  |
| --- | --- | --- | --- |
| Topic | Item | Checklist item description | Reported on Page |
| **Title** | **1** | The diagnosis or intervention of primary focus followed by the words “case report” | _1______________ |
| **Key Words** | **2** | 2 to 5 key words that identify diagnoses or interventions in this case report, including "case report" | _1______________ |
| **Abstract (no references)** | **3a** | Introduction: What is unique about this case and what does it add to the scientific literature? | _3______________ |
|  | **3b** | Main symptoms and/or important clinical findings | _3______________ |
|  | **3c** | The main diagnoses, therapeutic interventions, and outcomes | _3______________ |
|  | **3d** | Conclusion—What is the main “take-away” lesson(s) from this case? | _3______________ |
| **Introduction** | **4** | One or two paragraphs summarizing why this case is unique (**may include references**) | _4______________ |
| **Patient Information** | **5a** | De-identified patient specific information | _5______________ |
|  | **5b** | Primary concerns and symptoms of the patient | _5______________ |
|  | **5c** | Medical, family, and psycho-social history including relevant genetic information | _5______________ |
|  | **5d** | Relevant past interventions with outcomes | _5______________ |
| **Clinical Findings** | **6** | Describe significant physical examination (PE) and important clinical findings | _5–6____________ |
| **Timeline** | **7** | Historical and current information from this episode of care organized as a timeline | _5–7____________ |
| **Diagnostic Assessment** | **8a** | Diagnostic testing (such as PE, laboratory testing, imaging, surveys ) | _5–7____________ |
|  | **8b** | Diagnostic challenges (such as access to testing, financial, or cultural) | _N/A____________ |
|  | **8c** | Diagnosis (including other diagnoses considered) | _5______________ |
|  | **8d** | Prognosis (such as staging in oncology) where applicable | _N/A____________ |
| **Therapeutic Intervention** | **9a** | Types of therapeutic intervention (such as pharmacologic, surgical, preventive, self-care) | _5–7____________ |
|  | **9b** | Administration of therapeutic intervention (such as dosage, strength, duration) | _5–7____________ |
|  | **9c** | Changes in therapeutic intervention (with rationale) | _6–7____________ |
| **Follow-up and Outcomes** | **10a** | Clinician and patient-assessed outcomes (if available) | _6–7, 13_________ |
|  | **10b** | Important follow-up diagnostic and other test results | _6–7____________ |
|  | **10c** | Intervention adherence and tolerability (How was this assessed?) | _7______________ |
|  | **10d** | Adverse and unanticipated events | _5–7, 10_________ |
| **Discussion** | **11a** | A scientific discussion of the strengths AND limitations associated with this case report | _10_____________ |
|  | **11b** | Discussion of the relevant medical literature **with references** | _8–11___________ |
|  | **11c** | The scientific rationale for any conclusions (including assessment of possible causes) | _8–11, 12________ |
|  | **11d** | The primary “take-away” lessons of this case report (without references) in a one paragraph conclusion | _12_____________ |
| **Patient Perspective** | **12** | The patient should share their perspective in one to two paragraphs on the treatment(s) they received | _13_____________ |
| **Informed Consent** | **13** | Did the patient give informed consent? Please provide if requested | **YES** |
